# Multiplex Patient-Based Drug Response Assay in Pancreatic Ductal Adenocarcinoma

**DOI:** 10.3390/biomedicines9070705

**Published:** 2021-06-23

**Authors:** Andrew Armstrong, Muhammad R. Haque, Sina Mirbagheri, Usman Barlass, Douglas Z. Gilbert, Jaimin Amin, Ajaypal Singh, Ankur Naqib, Faraz Bishehsari

**Affiliations:** 1 Department of Internal Medicine, Division of Gastroenterology, Rush University Medical Center, Chicago, IL 60612, USA; armstrong.andrew7@gmail.com (A.A.); sina.mirbagheri@gmail.com (S.M.); Usman_Barlass@rush.edu (U.B.); dzgilbert@gmail.com (D.Z.G.); jaiminamin@gmail.com (J.A.); Ajaypal_Singh@rush.edu (A.S.); 2 Division of Digestive Diseases, Rush Center for Integrated Microbiome & Chronobiology Research, Rush University Medical Center, Chicago, IL 60612, USA; muhammad_r_haque@rush.edu (M.R.H.); ankur_naqib@rush.edu (A.N.)

**Keywords:** pancreatic ductal adenocarcinoma, patient-derived organoid, drug screening

## Abstract

Pancreatic ductal adenocarcinoma (PDA) is an extremely lethal malignancy arising from the pancreas. The treatment of PDA is complicated by ineffective treatments and a lack of biomarkers predictive of treatment success. We have designed a patient-derived organoid (PDO) based high-throughput drug screening assay to model treatment response to a variety of conventional and investigational treatments for PDA. Consecutive patients undergoing endoscopic ultrasound-guided fine-needle biopsy for tissue diagnosis of PDA at Rush University Medical Center were offered to participate in the study. Biopsies were immediately processed to develop organoids. Fifteen PDOs were screened for sensitivity to 18 compounds, including conventional PDA chemotherapies and FDA-approved investigational targeted therapies in cancer using Cell-titer GLO 3D (Promega) cell viability assay. The area under the curve (AUC) was calculated and normalized to the maximum area under the curve to generate a normalized AUC between 0 and 1. Molecular profiling of PDOs was conducted using RNA-seq. Human PDA transcriptomic was extracted from The Cancer Genome Atlas (TCGA). The drug response curves were reproducible. We observed variation in response to conventional therapies overall as well as among individual patients. There were distinct transcriptome signatures associated with response to the conventional chemotherapeutics in PDA. The transcriptomic profile of overall resistance to conventional therapies in our study was associated with poor survival in PDA patients in TCGA. Our pathway analysis for targeted drugs revealed a number of predictors of response associated with the mechanism of action of the tested drug. The multiplex organoid-based drug assay could be used in preclinical to inform patient stratification and therapeutic selection in PDA. When combined with omics data, ex vivo response to treatment could help identify gene signatures associated with response to novel therapies.

## 1. Introduction

With an average five-year survival of less than 10%, pancreatic ductal adenocarcinoma (PDA) is an extremely lethal malignancy [1]. The disease rate is increasing; it is estimated that PDA will rise from the third to the second leading cause of cancer-related death in the U.S. by 2025 [2]. Systemic therapies are often ineffective with high rates of intrinsic and acquired resistance to conventional treatment regimens. Predictive biomarkers to guide treatment selection and precision medicine-based treatment paradigms are desperately needed to combat this deadly disease. However, precision medicine-based clinical trials in PDA have proven to be expensive and ineffective. Inability to obtain surgical tissue at diagnosis, insufficient content of diagnostic biopsy for adequate sequencing depth, and deteriorating patient performance status prior to receiving treatment are all common reasons for patient exclusion [3,4,5]. The advent of three-dimensional organoids and patient-derived organoids (PDO) has resulted in a paradigm shift in how preclinical analyses can model tissues and cancers [6]. We and others have shown that organoids retain a high degree of similarity to the original tissue and the patient tumors, including PDA [7,8]. Recent studies using PDO models of pancreatic cancers have generated genomic signatures associated with sensitivity to chemotherapies [9]. Here, we have developed a multiplex drug screening assay to assess ex vivo drug response to conventional and investigational drugs in PDA and identify gene signature and pathways that may influence response to therapy.

## 2. Materials and Methods

### 2.1. Human Specimens

All pancreas cancer tissue was collected from patients undergoing endoscopic ultrasound and tissue biopsy at Rush University Medical Center. Written informed consent was collected from all patients prior to the collection of tissue. Tissue collection and experiments were reviewed and approved by the Institutional Review Board at Rush University.

### 2.2. Generation of Patient-Derived Organoids

Core biopsy tissue samples were collected from patients undergoing endoscopic ultrasound for clinical confirmation of PDA at Rush University Medical Center. Only samples confirmed to be PDA by pathological assessment were included in the study. Biopsies were processed immediately following sample acquisition and cultured for at least two passages prior to multiplex analysis. Biopsy samples were digested in digestion media: 1:3 ratio of Advanced DMEM/F12 (Invitrogen, Waltham, MA, USA) and TrypLE Express, supplemented with collagenase XI 0.012% (*w*/*v*) (Sigma-Aldrich, St. Louis, MO, USA), 0.012% dispase (*w*/*v*) (Gibco, Gaithersburg, MD, USA), 10.5 µM Y-27632 (Sellekchem, Houston, TX, USA), and 10 mg/mL DNAseI (Sigma-Aldrich, St. Louis, MO, USA) at 37 °C. Cells were plated in 50 µL domes into 24-well plates with Matrigel (Corning, Corning, NY, USA), and supplemented with organoid growth medium: Advanced DMEM/F12 (Invitrogen, Waltham, MA, USA), 1 M HEPES (Sigma-Aldrich, St. Louis, MO, USA), 1× B27-supplement (Invitrogen, Waltham, MA, USA), 1X N2-supplement (Invitrogen, Waltham, MA, USA), 1 mM *N*-Acetylcysteine (Sigma-Aldrich, St. Louis, MO, USA), 10 mM nicotinamide (Sigma-Aldrich, St. Louis, MO, USA), hGastrin 0.1 µmol/L, hEGF 50 ng/mL, 500 nM A83-01 (Sellekchem, Houston, TX, USA), Y-27632 (Sellekchem, Houston, TX, USA), 100 ng/mL hFGF-10 (PeproTech, Rocky Hill, NJ, USA), and Wnt3A-R-spondin1-Noggin condition media (50% of final volume). Organoids were routinely supplemented with fresh media and mechanically disassociated for expansion.

### 2.3. Histological Analysis of Patient-Derived Organoids

Organoids were recovered from Matrigel using cell recovery solution (Sigma-Aldrich, St. Louis, MO, USA) according to the manufacturer’s instructions. After recovery, organoids were fixed in 10% neutral buffered formalin. As previously described, organoids were suspended in 4% agarose prior to embedding in paraffin blocks [10]. Paraffin blocks were section and stained with hematoxylin and eosin.

### 2.4. Compound Selection

Selected compounds include conventional as well as investigation drugs for the treatment of PDA (Table 1). Conventional standard of care chemotherapeutics was selected based on the clinical practice guidelines of the National Comprehensive Cancer Networks (NCCN), American Society of Clinical Oncology, and the European Society for Medical Oncology [11,12,13]. Additional compounds were chosen based on targeting of frequently altered pathways in PDA as well as potential efficacy according to the available clinical trials in PDA. Compounds used were obtained through the NIH Developmental Therapeutics Program using the NCI-Approved Oncology Drug Set, with the exception of Napabucasin (Selleckchem, Houston, TX, USA).

### 2.5. Multiplex Drug Screening Assay

Organoids were disassociated into single cells and strained through a 40 µm cell strainer. One thousand cells were plated in 20 µL of 10% Matrigel/complete organoid media into the inside 308 wells of a 384-well plate and supplemented with 10 µL of complete organoid media. PDOs were then cultured for 48 h prior to the addition of therapeutic compounds. PDOs were tested by drug screening at similar passages. Compounds were diluted in DMSO via a 5-fold serial dilution from 1000 to 1.6 µM and further diluted 1:100 in complete organoid media to achieve a concentration range of 10 µM–16 nM. Compounds were tested using three replicates, with five concentrations per drug, and normalized to a 0.5% DMSO control. Viability was analyzed using Cell-titer GLO 3D (Promega, Germany) cell viability reagent optimized for 3D cultures according to the manufacturer’s instruction, and luminescence was measured on a Synergy HT plate reader. Organoid viability was analyzed in GraphPad Prism 8 and fit using three-parameter least-squares logistic regression. The area under the curve (AUC) was calculated and normalized to the maximum area under the curve to generate a normalized AUC between 0 and 1, as previously described [9]. Heatmaps were generated using the ComplexHeatmap R package and GraphPad Prism 9 [14].

### 2.6. RNA-Seq

To capture the transcriptomic signatures associated with ex vivo treatment, RNA was extracted from organoids at the time of multiplex analysis. RNA quality and quantity were measured using Agilent 4200 Tapestation using high Sensitivity RNA ScreenTape System (Agilent Technologies). Library preparation was completed using the SMARTer Stranded Total RNA-Seq Kit v2 (Takeda, Tokyo, Japan). Libraries were sequenced on an Illumina NextSeq 500 instrument (Illumina Inc., San Diego, CA, USA) using NextSeq 500 High Output reagent kit (Illumina Inc., San Diego, CA, USA) (1 × 75 cycles) with a target read depth of approximate (5–10) million aligned reads per sample. RNA-Seq read quality was quantified using FastQC [15]. Reads were aligned and mapped to the human genome GRCh38 using STAR, and Featurecounts were used to extract counts per gene [16,17]. Counts per gene were normalized using EdgeR [18]. Genes were filtered to include genes with at least one count in at last two samples. Clustering analysis was performed using K means clustering was in R using the stats (v3.6.2) package with 4 centers [19]. The Cancer Genome Atlas (TCGA) data were accessed using cBioPortal [20]. mRNA expression data and clinical data of 183 PDA samples were obtained from the TCGA pancreatic cancer cohort (TCGA-PAAD) database (https://portal.gdc.cancer.gov/ accessed on 26 May 2021). Survival Analysis and Kaplan–Meier plots were generated using GraphPad Prism 9 (San Diego, CA, USA). 

### 2.7. Pathways Analysis

Pathway analysis was conducted using Gene Set Enrichment Analysis of KEGG pathways based on RNAseq gene expression. Pathway analysis and ridge plots were generated using the ClusterProfiler R package [21]. Gene set enrichment subgroup analysis was conducted using GSEA Prerank of genes identified by Spearman rank correlation as being highly correlated to treatment response (Spearman rho < |0.6|) and FDR < 0.25. Positive pathways indicate pathways that are associated with drug resistance. Negative pathways indicate pathways that are associated with drug sensitivity.

## 3. Results

**Figure 1:**
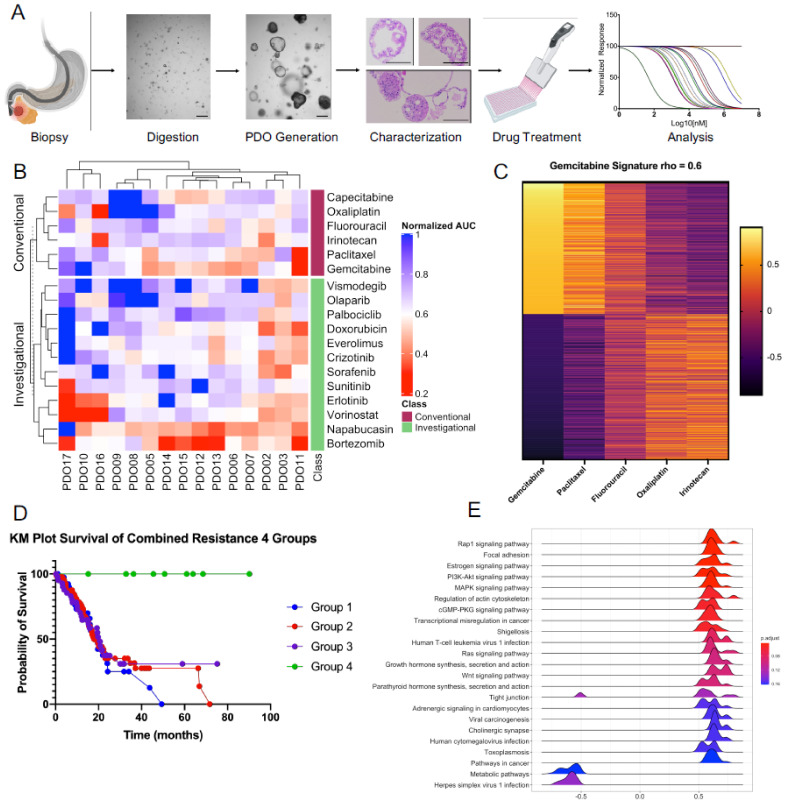
(**A**) Workflow describing the generation, characterization, and multiplex-drug screening assay of PDOs from EUS FNA biopsies (created with Biorender); scale bar 100 µm (**B**) Heatmap of normalized area under the curve for all drugs. Analysis reveals heterogeneity in PDO response, both within individual drug treatments and within PDOs. Normalize area under the curve between 0 and 1 (Red—smaller AUC; drug sensitivity, Blue—AUC; drug resistance); (**C**) Heatmaps of highly correlated gene clusters specific to drug response. For each drug, genes were filtered to include only genes with Spearman rho values rho > |0.6| generating drug-specific gene profiles highly correlated with treatment response. Spearman rho values for each drug profile were then compared between all conventional drugs. Similarities in drug profiles indicate that similar gene expression is correlated with drug response; (**D**) Kaplan–Meier plot of PDA patients clustered by expression of combined resistance signatures. Clusters are ordered by expression 1–4, 1 being the highest and 4 is the lowest; (**E**) Ridge plot of KEGG pathways associated with response to Palbociclib treatment. Positively enriched pathways are associated with treatment sensitivity, and negatively enriched pathways are associated with treatment resistance. Pathway analysis was conducted using Gene Set Enrichment Analysis of KEGG pathways based on RNAseq gene expression.

PDOs were generated using biopsy material collected from 20 PDA patients undergoing endoscopic ultrasound-guided fine-needle biopsy. Two patients were excluded as they did not have PDA per histology. Organoids did not grow from two samples, while the organoids from another sample did not propagate into enough organoids needed for multiplex drug testing. PDOs varied greatly in their morphology with a mix of cystic and dense structures with cystic ductal features. Hematoxylin and Eosin staining of the PDO sections revealed complex architecture and cellular atypia, including hyperchromatic nuclei and loss of cell polarization, consistent with histologic changes in PDA (Figure 1A). The PDA diagnosis was confirmed by a clinical pathologist upon review of the original biopsy. In total, 15 PDOs underwent drug assay to 18 drugs, including 6 conventional chemotherapies used in the treatment of PDA and a number of investigational drugs that are currently being evaluated in cancer clinical trials.

We observed intra-individual variations in PDOs response to the tested drugs as well as inter-individual variations in response to each treatment (Figure 1B; Supplemental Figure S1A). Importantly, we observed that PDOs retain their relative drug sensitivity upon repeat testing by the same drug, indicating reproducibility of our drug assay (Supplemental Figure S1B).

The observed heterogeneity among PDOs in their overall drug sensitivity is consistent with the existing clinical data that indicate variation in response to therapy, which could be related to heterogeneity in tumor molecular profile among PDA patents [22,23].

To identity gene signature and pathways that could predict drug response ex vivo, we performed RNA-seq on the collected RNA from PDOs at the time of drug assay. Currently recommended first-line systemic treatments for PDA include FOLFIRINOX (fluorouracil, oxaliplatin, irinotecan) or gemcitabine plus paclitaxel. Recent clinical data show a patient-specific pattern in response to either of the two major conventional PDA chemotherapeutics, suggesting there may be tumor profiles predictive of response to one and not the other chemotherapy [24]. In order to find a possible pattern of a gene signature that may correlate with response to conventional chemotherapies in our study, individual genes were associated with treatment response using Spearman’s correlation. Genes were deemed to be associated with treatment response if Spearman rho values were <|0.6| (Figure S3). Genes with negative rho values are associated with sensitivity to treatment and positive rho values associated with treatment resistance. Interestingly, genes associated treatment response cluster within systemic treatment regimens (FOLFIRINOX vs. Gem + Pac) (Figure 1C). For example, the gene signature associated with gemcitabine sensitivity was most closely related to paclitaxel, while genes associated with sensitivity to Irinotecan were most similar to oxaliplatin and least similar to gemcitabine. In our relatively small cohort, it appears that each PDA regimen is associated with a unique gene signature distinct from the other regimen and that response to one regimen is not associated with response to the other. In order to see if our gene signature could be relevant to the larger cohorts of PDA, we extracted transcriptomic data from RNA-seq analyses of patients with PDA diagnosis included in TCGA.

We then curated genes associated with resistance to all conventional chemotherapeutics in our study. To this end, we identified genes associated with ex vivo resistance to all the six conventional drugs (defined as having a spearman rho < 0.8) in our series to create a “resistance signature”. Using this resistance signature, patients from the TCGA-PAAD database were clustered into four groups. Analysis of survival outcomes from TCGA clinical data revealed that low expression of our resistance signature was associated with increased survival of the PDA cohort (Figure 1D).

The development of therapies that target specific pathways brought promises of improving treatment efficacy while decreasing the side effects of conventional chemotherapies in cancer. The disappointing response rate of targeted therapies in clinical trials is partly due to the lack of predictive biomarkers of drug response or a relatively low number of patients with a specific molecular profile who would potentially benefit from the tested targeted therapy in a clinical trial [25]. To identify pathway-based predictive biomarkers of response to the targeted therapies in our assay, we performed gene set enrichment analysis to identify pathways based on the genes associated with sensitivity to each of the investigational targeted therapies (Figure S4). Analysis of pathways associated with resistance to Palbociclib, a CDK 4/6 cell cycle inhibitor, revealed multiple growth and signaling pathways (i.e., PI3K, MAPK, Rap1, and Ras) (Figure 1E) that predicted response to this drug. Pathways associated with response to other targeted therapies are shown in Supplemental Figures S2 and S3. For example, the ERBB1 downstream pathway was identified to be associated with sensitivity to ERBB1 inhibitor, erlotinib. The PARP inhibitor olaparib is shown to be associated with multiple pathways of DNA damage repair and chromosome organization (Supplemental Figure S2).

## 4. Discussion

We have designed a patient-based multiplex drug screening assay using patient-derived organoids (PDOs) for the assessment of ex vivo drug response to conventional PDA treatments as well as targeted therapies. We observed an overall ex vivo efficacy of conventional chemotherapeutics on PDA organoids. Consistent with the reported heterogeneity in clinical response to therapies among PDA patients, we found variations in ex vivo response to the conventional therapies in each patient and to each treatment among different patients. Our simultaneous transcriptomics helped us identify possible patterns and predictors of response.

Recent publications have highlighted the utility of PDOs in biomarker development in PDA [26]. Indeed, organoids are proving to be rapid and effective models of human cancers capable of recapitulating drug response and inter-patient genetic heterogeneity [27]. Our success in developing and propagating enough PDOs from the collected biopsies (15/18, >80%) is acceptable and consistent with other reports [28,29]. As expected, PDOs displayed significant heterogeneity in overall response and response to individual treatments.

FOLFIRINOX and gemcitabine plus nab-paclitaxel are the two conventional systemic regimens currently recommended and used as first-line chemotherapeutic regimens in PDA. There is currently no molecular stratification tool for selecting the choice of initial treatment in PDA patients. In the absence of a head-to-head trial testing on the comparative efficacy of the two regimens, the treatment choice is practically made according to the clinician choice and the patient’s tolerance. Clinical observations suggest a subset of patients who do not show response to one regimen may respond to the alternative regimen [30]. In the current preclinical study of ex vivo drug response in our prospectively collected samples of PDA, we found that transcription signature associated with response to drugs as part of FOLFIRINOX and gemcitabine-based regimens could be different. Drugs within each regimen appeared to share similar transcriptomic signatures associated with treatment response, while this was not the case when comparing signatures between drugs of different regimens. These findings suggest that there may exist a molecular signature that could predict response to either of the approved first-line treatments in PDA and that lack of response to one regimen should not necessarily predict the lack of efficacy of the second regimen. The latter is consistent with studies aimed at establishing second-line therapies in PDA, which have shown gemcitabine plus paclitaxel was clinically beneficial following progression on first-line FOLFIRINOX [30].

Unlike many other cancers, no clinically relevant molecular classification system has been developed for PDA [31]. A study by Tiriac et al. showed that patient-derived organoids could be used to identify transcriptomic signatures indicative of response to chemotherapy [9]. Similarly, in our study, we were able to generate a transcriptomic signature associated with resistance to conventional therapies. In addition, we were able to show that low expression of this “resistance” signature was associated with greater survival in PDA patients. These could have implications in the development of preclinical biomarkers to inform patient prognosis and help clinical decision-making about patients with a high likelihood of resistance to conventional therapies for possible inclusion in clinical trials using investigational targeted therapies. Our results also suggest that response to targeted agents could be informed by pathway-based transcriptomic signatures of the PDOs.

Our gene set enrichment analysis identified a number of predictive pathways of ex vivo drug response that were associated with the mechanism of actions of several targeted therapies. Our analysis of Palbociclib identified multiple pathways associated with treatment resistance, including PI3K. Activation of PI3K is associated with Palbociclib resistance in pancreatic and other cancers [32,33]. Palbociclib was also shown to work in synergy with multiple PI3K/mTOR inhibitors in pancreatic cancer cell lines [34]. Similarly, pathways relevant to erlotinib and olaparib were associated with sensitivity to these drugs. The clinical utility of olaparib and other PARP inhibitors in HR deficient cancers highlights the potential of personalized medicine in cancer treatment. The development of BRCAness signatures and homologous recombination deficiency test have proven to be important predictors of response to therapy in multiple cancers [35,36,37]. Association of pathways related to the mechanism of a number of targeted agents with response to those agents in our relative sample size supports the potential utility of patient derided organoids as a preclinical model in the development of biomarkers for personalized medicine. Larger multi-center studies are needed to identify comprehensive molecular profiles of drug response, which could be potentially used for agent selection in clinical trials in PDA. It is worth noting that several limitations of our study have been identified and will need to be addressed in future studies. Our study could not verify ex vivo response to treatment with patient treatment data. A number of patients opted for palliative care, and with the limited number of patients remaining, we did not have enough data to validate treatment response. In addition, we did perform exome sequencing and were unable to generate data regarding the mutational status of key PDA driver genes or our PDOs.

## 5. Conclusions

Our data support the continued study of PDOs to investigate the response to conventional and investigational treatment of PDA. Our results contribute to the growing body of evidence that PDOs are useful preclinical models of drug response in pancreatic cancer. When combined with omic data, ex vivo response to treatment may be beneficial in identifying gene signatures associated with response to the agent. Such data have the potential to inform patient stratification and therapeutic selection.

## Figures and Tables

**Table 1:** Compounds included in multiplex PDO-based drug screening platform. Conventional therapies for the treatment of PDA, according to the National Comprehensive Cancer Network (NCCN), are highlighted in gray.

Drug Name (USAN)	WHO ATC Code	WHO Drug Class	Mechanism of Action	Targets	FDA Status	
**Fluorouracil**	L01BC02	Nucleoside Metabolic Inhibitor [EPC]	Nucleic Acid Synthesis Inhibitors [MoA]	Conventional chemotherapy	Approved	**Conventional**
**Gemcitabine**	L01BC05	Nucleoside Metabolic Inhibitor [EPC]	Nucleic Acid Synthesis Inhibitors [MoA]	Conventional chemotherapy	Approved	
**Capecitabine**	L01BC06	Nucleoside Metabolic Inhibitor [EPC]	Nucleic Acid Synthesis Inhibitors [MoA]	Conventional chemotherapy	Approved	
**Paclitaxel**	L01XX19	Microtubule Inhibitor [EPC]	Microtubule Inhibition [PE]	Conventional chemotherapy	Approved	
**Oxaliplatin**	L01XA03	Platinum-based Drug [EPC]	DNA Damaging Agent	Conventional chemotherapy	Approved	
**Irinotecan**	L01CD01	Topoisomerase Inhibitor [EPC]	Topoisomerase Inhibitors [MoA]	Conventional chemotherapy	Approved	
**Erlotinib**	L01XE03	Kinase Inhibitor [EPC]	Protein Kinase Inhibitors [MoA]	EGFR, PTPRF	Approved	**Investigational**
**Sunitinib**	L01XE04	Kinase Inhibitor [EPC]	Protein Kinase Inhibitors [MoA]	FGFR1, FLT3, FLT4, PDGFRB, FLT1	Approved	
**Sorafenib**	L01XE05	Kinase Inhibitor [EPC]	Protein Kinase Inhibitors [MoA]	BRAF, RAF1, FLT4, KDR, FLT3	Approved	
**Everolimus**	L01XE10	mTOR Inhibitor Immunosuppressant [EPC]	mTOR Inhibitors [MoA]	MTOR, AKT1, AKT2, AKT3, FKBP1A	Approved	
**Bortezomib**	L01XX32	Proteasome Inhibitor [EPC]	Proteasome Inhibitors [MoA]	PSMA6, PSMA7, PSMB2, PSMB5, PSMB1	Approved	
**Vorinostat**	L01XX38	Histone Deacetylase Inhibitor [EPC]	Histone Deacetylase Inhibitors [MoA]	HDAC1, HDAC10, HDAC11, HDAC2, HDAC3	Approved	
**Vismodegib**	L01XX43	Hedgehog Pathway Inhibitor [EPC]	Smoothened Receptor Antagonists [MoA]	SMO, PTCH1	Approved	
**Olaparib**	L01XX46	Poly(ADP-Ribose) Polymerase Inhibitor [EPC]	Poly(ADP-Ribose) Polymerase Inhibitors [MoA]	PARP1, PARP2, PARP3, BRCA2, PIK3CA	Approved	
**Doxorubicin**	L01DB01	Anthracycline Topoisomerase Inhibitor [EPC]	Topoisomerase Inhibitors [MoA]	TOP2A, KRT20	Approved	
**Crizotinib**	L01XE16	Kinase Inhibitor [EPC]	Receptor Tyrosine Kinase Inhibitors [MoA]	ROS1, ALK, MET, ERBB2, ABL1	Approved	
**Palbociclib**	L01XE33	Kinase Inhibitor [EPC]	Kinase Inhibitors [MoA]	CDK4, CDK6, DRD2, DRD4, CCND1	Approved	
**Napabucasin**	NA	Naphthofurans [EPC]	STAT3 Inhibitor [MoA]		Approved	

## Data Availability

The data sets generated for this study can be found in the BioProject database (BioProject ID: PRJNA732905, https://www.ncbi.nlm.nih.gov/bioproject/732905 accessed on 26 May 2021).

## Supplementary Materials

The following are available online at https://www.mdpi.com/article/10.3390/biomedicines9070705/s1, Figure S1: PDO Viability, Figure S2: GSEA Pathway Analysis, Figure S3: Heatmaps of genes highly correlated with drug sensitivity, Figure S4: Ridge plot of KEGG pathways associated with response to individual drugs.
