# Supplementary material for: Multiplex Patient-Based Drug Response Assay in Pancreatic Ductal Adenocarcinoma
Source: Biomedicines. 2021 Jun 23;9(7):705. doi: 10.3390/biomedicines9070705 (PMC8301364; doi:10.3390/biomedicines9070705)
Supplement: Supplementary file 1 [file biomedicines-09-00705-s001.zip › biomedicines-1239007-supplementary.pdf]

biomedicines

MDPI

Article

Multiplex Patient-Based Drug Response Assay in Pancreatic Ductal Adenocarcinoma

Andrew Armstrong [1], Muhammad R. Haque [2], Sina Mirbagheri [1], Usman Barlass [1], Douglas Z. Gilbert [1], Jaimin Amin [1], Ajaypal Singh [1], Ankur Naqib [2] and Faraz Bishehsari [2,*]

Supplementary materials

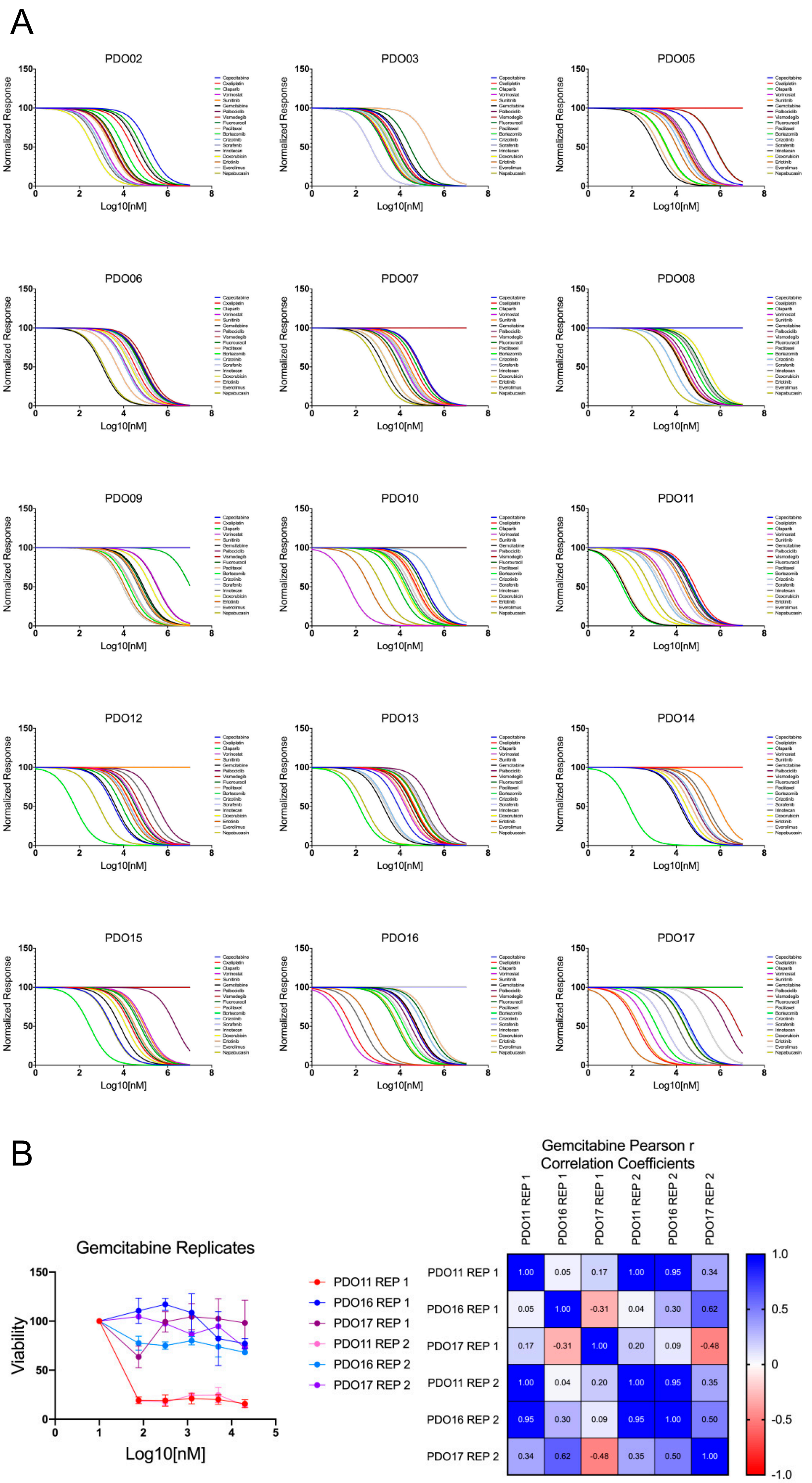
A
PDO02
PDO03
PDO05
PDO06
PDO07
PDO08
PDO09
PDO10
PDO11
PDO12
PDO13
PDO14
PDO15
PDO16
PDO17
Normalized Response
Log10[nM]
B
Gemcitabine Replicates
Viability
Log10[nM]
PDO11 REP 1
PDO16 REP 1
PDO17 REP 1
PDO11 REP 2
PDO16 REP 2
PDO17 REP 2
Gemcitabine Pearson r
Correlation Coefficients
PDO11 REP 1 1.00 0.05 0.17 1.00 0.95 0.34
PDO16 REP 1 0.05 1.00 -0.31 0.04 0.30 0.62
PDO17 REP 1 0.17 -0.31 1.00 0.20 0.09 -0.48
PDO11 REP 2 1.00 0.04 0.20 1.00 0.95 0.35
PDO16 REP 2 0.95 0.30 0.09 0.95 1.00 0.50
PDO17 REP 2 0.34 0.62 -0.48 0.35 0.50 1.00

Figure S1. (**A**) Viability curves of patient derived organoids for each drug. Lines represent non-linear regression curve fit based on viability data. Curves were set to a range of 0-7.(**B**) Co(rrelations curves depicting replicate drug screening assays for three individual PDOs. Replicate drug screens were repeated at least 2 weeks apart.

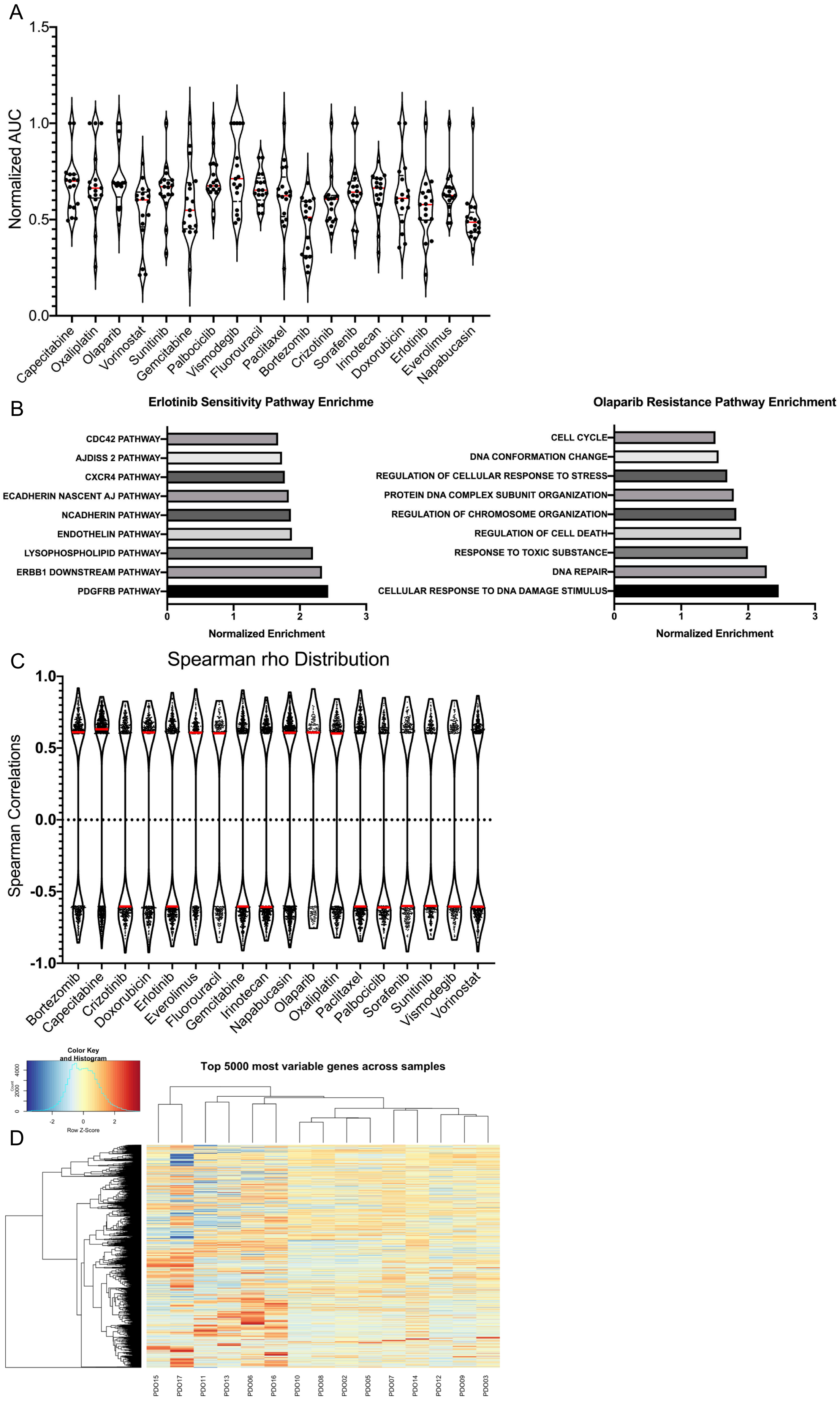
A
Normalized AUC
0.0
0.5
1.0
1.5
Capecitabine
Oxaliplatin
Olaparib
Vorinostat
Sunitinib
Gemcitabine
Palbociclib
Vismodegib
Fluorouracil
Paclitaxel
Bortezomib
Crizotinib
Sorafenib
Irinotecan
Doxorubicin
Erlotinib
Everolimus
Napabucasin
B
Erlotinib Sensitivity Pathway Enrichme
CDC42 PATHWAY
AJDISS 2 PATHWAY
CXCR4 PATHWAY
ECADHERIN NASCENT AJ PATHWAY
NCADHERIN PATHWAY
ENDOTHELIN PATHWAY
LYSOPHOSPHOLIPID PATHWAY
ERBB1 DOWNSTREAM PATHWAY
PDGFRB PATHWAY
Normalized Enrichment
Olaparib Resistance Pathway Enrichment
CELL CYCLE
DNA CONFORMATION CHANGE
REGULATION OF CELLULAR RESPONSE TO STRESS
PROTEIN DNA COMPLEX SUBUNIT ORGANIZATION
REGULATION OF CHROMOSOME ORGANIZATION
REGULATION OF CELL DEATH
RESPONSE TO TOXIC SUBSTANCE
DNA REPAIR
CELLULAR RESPONSE TO DNA DAMAGE STIMULUS
Normalized Enrichment
C
Spearman rho Distribution
Spearman Correlations
1.0
0.5
0.0
-0.5
-1.0
Bortezomib
Capecitabine
Crizotinib
Doxorubicin
Erlotinib
Everolimus
Fluorouracil
Gemcitabine
Irinotecan
Napabucasin
Olaparib
Oxaliplatin
Paclitaxel
Palbociclib
Sorafenib
Sunitinib
Vismodegib
Vorinostat
Color Key and Histogram
Row Z-Score
Top 5000 most variable genes across samples
D
PDO15
PDO17
PDO11
PDO13
PDO06
PDO16
PDO10
PDO08
PDO02
PDO05
PDO07
PDO14
PDO12
PDO09
PDO03

Figure S2. (**A**) The distribution of normalized AUC values for all drugs. (Mean; red line, quartiles; dashed gray lines) (**B**) Gene set enrichment analysis of drug specific gene profiles reveals pathways correlated with erlotinib and olaparib sensitivity. Pathways are plotted using normalized enrichment scores and all pathways are significant and FDR Q < 0.25. (**C**) The distribution of Spearman correlation coefficient for each drug. Using gene expression and treatment response for all PDOs, the spearman rank correlation of gene expression to normalized AUC were calculated for each gene. (**D**) Heatmap and histogram of the top 5000 most variably expressed genes among all PDOs.

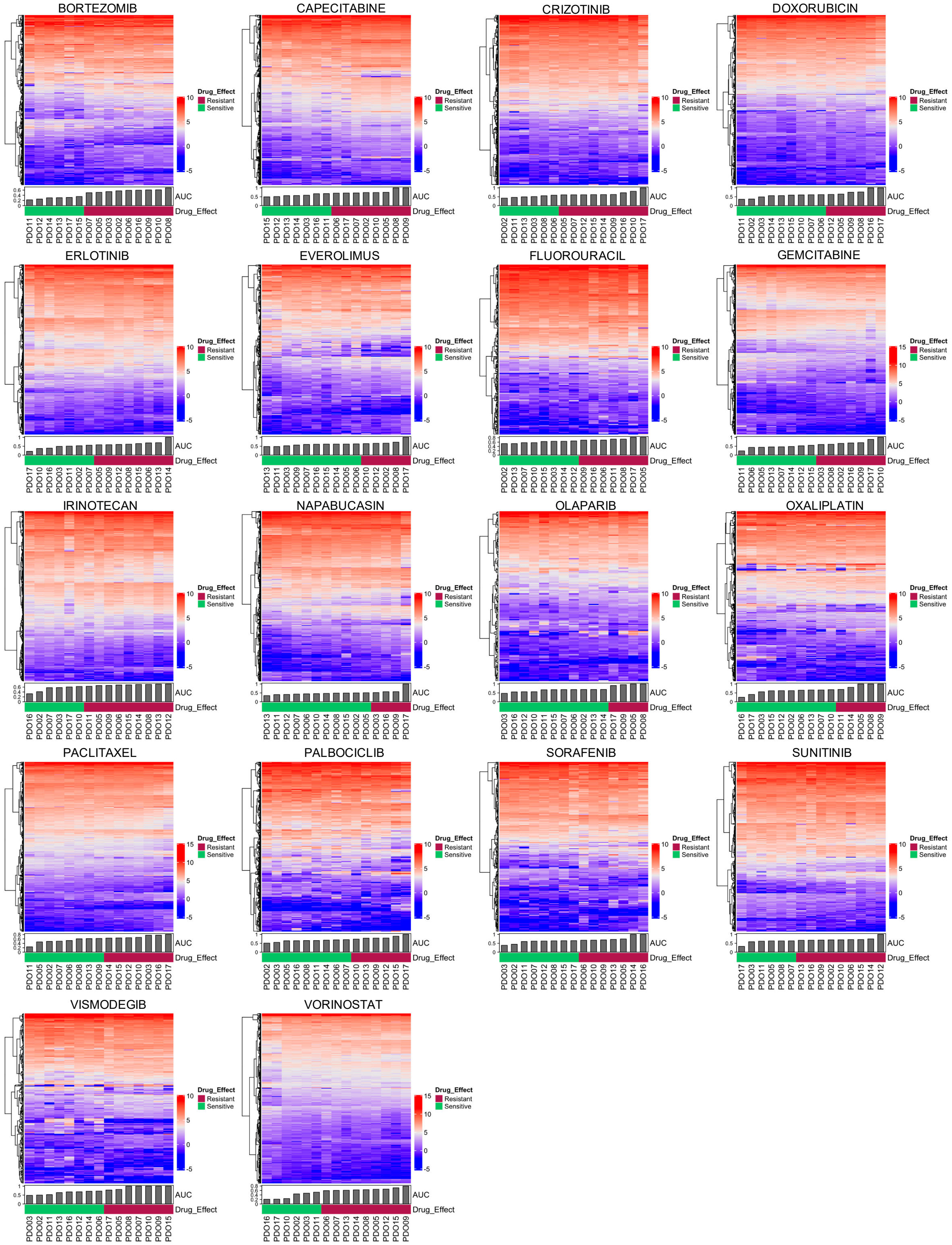

Figure S3. Heatmaps of log 2 cpm gene expression for genes that are highly correlated with drug sensitivity. Bar plots indicate normalize AUC for each PDO. Normalized AUC values were transformed to z-scores and PDOs with negative z-scores were specified as resistant to drug treatment (Green-sensitive, Maroon-resistant). Highly correlated genes–rho > |0.6|.

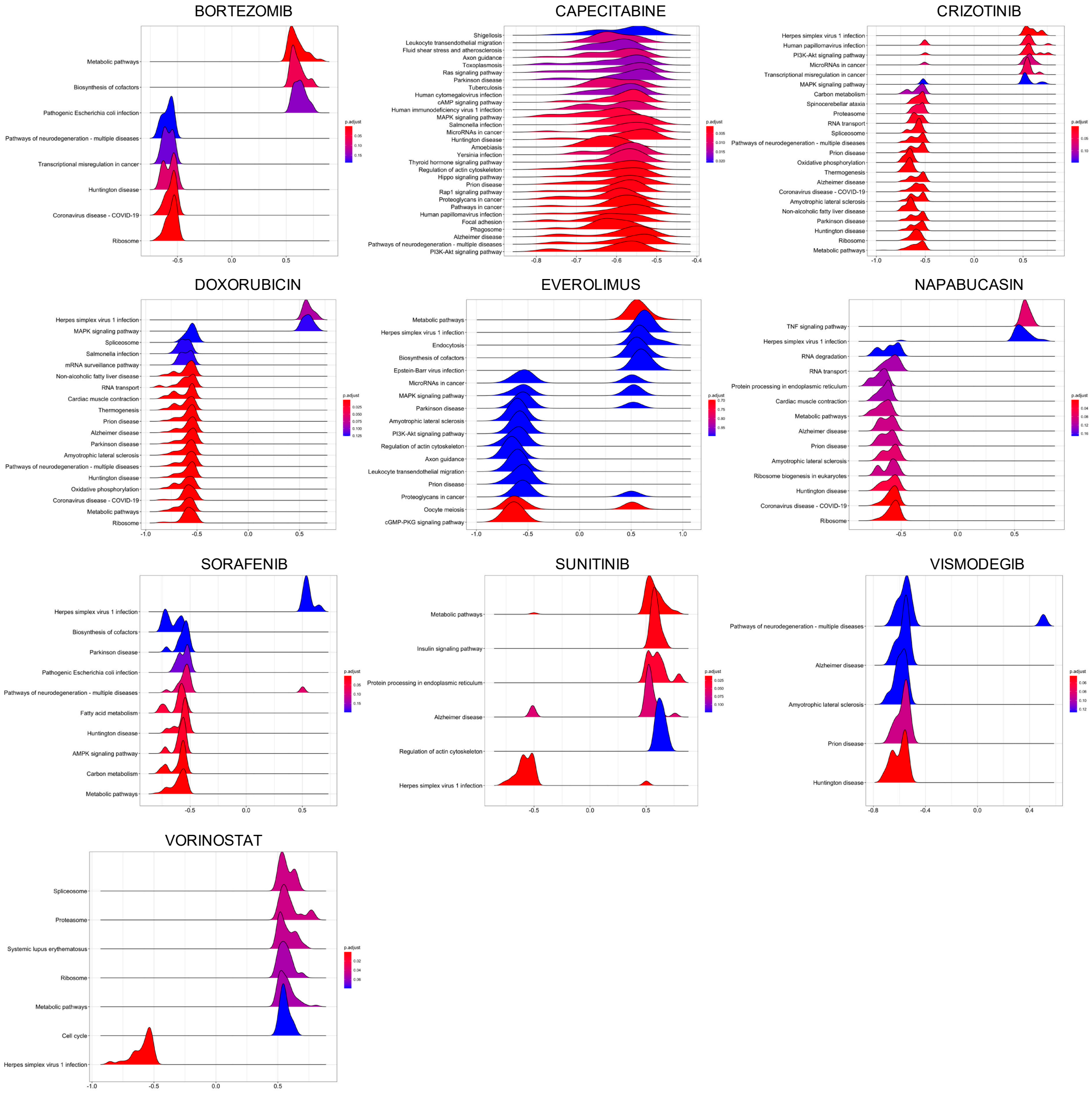

Figure S4. Ridge plot of KEGG pathways associated with response to individual drugs. Positively enriched pathways are associated with treatment sensitivity, and negatively enriched pathways are associated with treatment resistance. Pathway analysis was conducted using Gene Set Enrichment Analysis of KEGG pathways based on RNAseq gene expression.
